# Speech and Burden of Secondary Surgical Interventions Following One-Stage Repair of Unilateral Cleft Lip and Palate and Alveolar Bone Grafting Performed at Different Timings

**DOI:** 10.3390/jcm12175545

**Published:** 2023-08-25

**Authors:** Andrzej Brudnicki, Elżbieta Radkowska, Ewa Sawicka, Piotr Stanisław Fudalej

**Affiliations:** 1 Department of Maxillo-Facial Surgery, Pediatric Surgery Clinic, Institute of Mother and Child, Kasprzaka Str. 17a, 01-211 Warsaw, Poland; ewa.sawicka@imid.med.pl; 2 Speech and Language Pathology Clinic, Institute of Mother and Child, Kasprzaka 17a, 01-211 Warsaw, Poland; elzbieta.radkowska@imid.med.pl; 3 Department of Orthodontics and Dentofacial Orthopedics, University of Bern, Freiburgstrasse 7, 3010 Bern, Switzerland; piotr.fudalej@unibe.ch; 4 Department of Orthodontics, Institute of Dentistry and Oral Sciences, Palacky University Olomouc, Palackého 12, 779 00 Olomouc, Czech Republic; 5 Department of Orthodontics, Jagiellonian University in Krakow, Montelupich 4, 31-155 Krakow, Poland

**Keywords:** cleft lip and palate, long term, one-stage repair, speech

## Abstract

A comprehensive assessment of the treatment outcome in cleft lip and palate involves evaluating speech and the impact of speech-correcting surgical interventions. This retrospective case–control study compared the speech outcomes of 37 boys and 19 girls with unilateral cleft lip and palate (UCLP) who underwent one-stage cleft repair at an average age of 8.1 months and alveolar bone grafting either before or after 6 years of age, with a non-cleft control group at an average age of 10 years. Two experienced speech and language pathologists conducted perceptual speech assessments using a specialized test of 27 sentences designed for Polish-speaking cleft patients. The results revealed that 5.3% had severe hypernasality, 1.8% had severely impaired speech intelligibility, 10.7% exhibited retracted compensatory articulations, and 7.1% displayed facial grimacing. Mild hyponasality was observed in 12.3% of patients, while 16.1% exhibited voice abnormalities. Additionally, 12.5% of patients required orofacial fistula repairs, 3.6% underwent pharyngoplasties, and 28.6% received ear ventilation tube insertions. The study indicates that speech abnormalities in UCLP patients were relatively infrequent and not highly severe, suggesting that the primary UCLP repair method presented effectively reduced the need for further surgical interventions, leading to positive speech outcomes.

## 1. Introduction

Several review articles [1,2,3] have suggested that speech dissatisfaction can be a significant issue for young people with cleft palate with or without cleft lip (CP ± L). This dissatisfaction appears to be more prevalent among post-adolescent patients compared to preadolescent ones [1,4], indicating a possible relationship with the developmental stage. Regardless of the phase of development, speech dissatisfaction can have negative impacts on various aspects of psychosocial functioning, including self-esteem, mental health, and quality of life [2]. This implies that teenagers who are dissatisfied with their speech may experience lower self-esteem, a higher risk of developing depression, or a poorer quality of life. Therefore, it is essential to assess speech development when evaluating the overall outcome of a cleft center’s treatment protocol.

One important parameter to consider in assessing speech in patients with CP ± L is its understandability. Understandability can be strongly influenced by hypernasality and uncontrolled nasal air emissions (NAEs), which are speech defects resulting from velopharyngeal insufficiency (VPI) and the presence of oronasal fistulas (ONFs), respectively. These problems are caused by an inadequate primary repair of the cleft. Correcting hypernasality and uncontrolled NAEs at early stages of speech development is crucial to avoid subsequent compensatory articulations and achieve better final speech results. Initiating alveolar bone grafting (ABG) at an earlier stage offers the advantage of combining speech-related surgical interventions, such as the closure of oronasal fistulas (ONFs) and pharyngoplasty (if deemed necessary), within the same surgical session. This approach consequently leads to a reduction in the total number of surgeries and hospital stays required throughout the treatment protocol. Additionally, performing these interventions before the age of 3–4 years ensures that patients are unlikely to retain memories of the procedures due to what is commonly referred to as infantile amnesia or childhood amnesia. This memory gap is crucial as it prevents potentially traumatic experiences from contributing to a heightened sense of cleft stigma later in the lives of the patients.

A comprehensive assessment of speech outcomes following a particular treatment protocol should include an evaluation of secondary surgical interventions aimed at correcting speech issues. Additionally, hearing problems resulting from Eustachian tube dysfunction [5] and associated with the insertion of a middle ear ventilation tube (VT) must be considered during speech evaluations. In patients with CP ± L, VTs are inserted to prevent hearing impairment caused by otitis media with effusion (OME) [6,7]. However, VT insertion to drain an affected ear can frequently lead to serious complications [8,9], especially in children with cleft defects [7] or those who require repeated VT insertions [10]. Therefore, the current recommendation is to insert a VT when the spontaneous resolution of OME in a child with cleft palate is unlikely (e.g., type B tympanogram or persistence for ≥3 months) [11].

Since the late 1980s, our protocol at the Warsaw Cleft Center, Institute of Mother and Child (IMC), Warsaw, Poland, has entailed conducting cleft closure surgery in the first year of life for all patients with unilateral cleft lip and palate (UCLP). Secondary repairs (if needed) and alveolar bone grafting (ABG) were subsequently performed (ABG usually between 8 and 11 years of age). The surgical approach to palatoplasty has undergone several changes over time. The classical bilateral von Langenbeck method was initially succeeded by the unilateral von Langenbeck method. Subsequently, single-layer vomeroplasty was introduced. The impact of these alterations on speech development has been previously evaluated [12,13]. Notably, an increased occurrence of oronasal fistulas (ONFs) was observed among children who underwent single-layer vomeroplasty [13]. This observation prompted further enhancements to the surgical technique. These modifications encompassed the incorporation of a double-layer closure for the hard palate, shorter lateral incisions, and more meticulous suturing of lateral incisions to mitigate scar formation. Additionally, adjustments were made to the timing of ABG. Specifically, patients who underwent one-stage cleft closure before 1998 underwent ABG between 8 and 11 years, while those who underwent one-stage closure in or after 1999 underwent ABG prior to 6 years of age. The rationale for the earlier ABG timing was to allow for an easier integration of the grafted bone in the alveolus due to potentially better regenerative abilities at a younger age. Moreover, early ABG provided an opportunity to close ONFs, which could improve speech production.

To our knowledge, the existing literature lacks information regarding the correlation between speech outcomes and the timing of ABG. Therefore, the overall objective of this study was to evaluate the speech outcomes and the need for secondary speech-related surgical interventions in patients with UCLP who underwent one-stage closure of the entire cleft with early ABG (i.e., before 6 years of age) compared to late ABG (i.e., after 6 years of age).

## 2. Materials and Methods

### 2.1. General Considerations

The present study received ethical approval from the institutional ethics committee (The Bioethics Committee of the IMC, reference 31/2016). The study adhered to the principles outlined in the Declaration of Helsinki. The surgeons responsible for the primary surgical cleft repair in patients from the study group were not involved in any part of this evaluation.

### 2.2. Speech-Related Clinical Management

As part of our standard protocol, all patients with UCLP treated at IMC underwent comprehensive multidisciplinary follow-up evaluations at the ages of 5, 10, 15, and 18 years. Audio and video recordings were mandatory for speech documentation during these time points. In cases where patients showed signs of VPI following primary palatoplasty, the examination was expanded with video nasoendoscopy. For such cases, intensive speech therapy focused on improving velopharyngeal sphincter function was applied for up to 2 years, followed by close speech observation. If speech did not significantly improve during this period, it strongly indicated the need for pharyngoplasty or sphincteroplasty based on the results of phoniatric evaluation using video nasoendoscopy. The decision regarding the timing and type of corrective procedure was always made collectively by a team of professionals experienced in cleft therapy, including surgeons, speech and language pathologists, and phoniatrists.

It was preferred to eliminate ONFs before the completion of the speech formation period to avoid any negative impact on speech development. Therefore, both ONF repair and VPI surgical correction were routinely scheduled before the age of 6. Prophylactic middle ear ventilation tube (VT) insertion was not performed in any of the patients. This procedure was only carried out if persistent symptoms of otitis media with effusion (OME) appeared. Audiograms were regularly conducted to monitor the hearing of each cleft patient by an audiologist. Typically, hearing was assessed using tympanometry at 6–8 weeks and 3–4 months after one-stage repair of the cleft, and then preferably once a year. Special emphasis was placed on checking the hearing status at the ages of 5 and 10 years. Additionally, patients underwent otoscopic examination and auditory function evaluation using brainstem-evoked response audiometry (BERA) before surgery and 3–4 months postoperatively. The need for subsequent BERA examinations was determined on an individual basis by an audiologist. Patients with any hearing problems were closely monitored by otological and audiological specialists.

### 2.3. UCLP Group

This retrospective study analyzed medical documentation collected at approximately 10 years of age. The included patients were consecutively treated, non-syndromic individuals with complete UCLP who underwent surgery between 7 April 2005 and 14 March 2007. Preoperative infant orthopedics (IO) was not employed in any of the patients. The surgical procedure for primary cleft repair followed a one-stage protocol using the same technique, which has been the standard method of surgical treatment for this cleft subtype at IMC. The surgical repair involved soft and hard palate repair, as well as cleft lip repair, during a single operation. The procedure has been described in detail in a recent publication [14].

The following data were extracted from the medical charts: gender, cleft subtype and extent, age at the one-stage repair of UCLP, any postoperative complications reported during follow-up appointments, information about subsequent cleft-related surgical procedures, and details regarding speech therapy (e.g., age at initiation, duration, intensity, results of audiological control tests). Special attention was given to information regarding the need for any secondary speech-related surgical interventions, such as VPI repair, ONF repair, VT insertions, or any other potential corrections performed up to the time of evaluation.

### 2.4. Control Group

Fifty randomly selected 10-year-old individuals (19 boys and 31 girls) without hearing problems, who spoke the same language and had the same ethnic background as the UCLP group, were included as the control group. None of the children had ONF or VT insertions. The mean age of the control group at the time of speech examination was 9.9 years (SD 0.9; range 9.0–11.7).

### 2.5. Speech Examination in UCLP Group

Prior to the speech examination, information regarding previous speech therapy, hearing potential, upper airway infections, and mode of breathing was noted. The speech evaluation adhered to standardized procedures, following the guidelines outlined below: a thorough physical examination and a perceptual speech assessment (PSA) were carried out independently by two experienced speech and language pathologists with expertise in cleft therapy. The PSA is a technique that engages trained evaluators, typically speech-language pathologists who listen to and evaluate diverse aspects of a patient’s speech, encompassing elements like articulation, phonology, voice quality, fluency, and overall speech clarity.

Video recordings were captured using a digital video camera recorder, the DCR-SR75E (Sony Corporation, Tokyo, Japan, 2008), and were subsequently subjected to processing. The speech analysis was based on a standardized speech test, comprising 27 sentences specifically designed for assessing patients with orofacial clefts and suitable for the Polish language inventory [15].

The following speech abnormalities were evaluated based on the video recordings: intelligibility (rated on a 5-point scale from 1 to 5), hypernasality (rated on a 3-point scale from 0 to 2), hyponasality (rated on a 3-point scale from 0 to 2), audible nasal emission (ANEs) (rated on a 2-point scale as present or absent), misarticulations (rated on a 2-point scale as present or absent), compensatory articulations (rated on a 2-point scale as present or absent), voice abnormalities (rated on a 2-point scale as present or absent), compensatory facial grimacing (rated on a 3-point scale from 0 to 2), and oral breathing (rated on a 2-point scale as present or absent).

### 2.6. Method Reliability

Intra- and inter-rater reliability were calculated to determine the agreement between the first and second assessments conducted by two speech pathologists experienced in treating cleft patients. The assessments were performed at least 1 month apart and involved 17 patients with UCLP, representing 30% of the UCLP group. A one-month timeframe for re-assessment strikes a balance between mitigating the memory effect and avoiding prolonged delays that could potentially impact the study’s overall timeline.

### 2.7. Statistical Analysis

Intra- and inter-rater reliability were assessed using kappa statistics (Cohen’s Kappa) and interpreted according to the following levels of agreement: poor agreement when κ < 0.20, fair agreement when κ = 0.20 to 0.40, moderate agreement when κ = 0.40 to 0.60, good agreement when κ = 0.60 to 0.80, and very good agreement when κ = 0.80 to 1.00.

All data were analyzed using Stata IC v.13 software (StataCorp, College Station, TX, USA). Descriptive statistics including mean, standard deviation (SD), median, quartiles, range, and frequency tables were calculated. Inter-group differences were assessed using tests of proportions or Wilcoxon rank-sum tests, as applicable. Pairwise correlations, with and without Bonferroni-adjusted significance levels, were computed between speech outcomes and demographic data such as age at cleft repair, age at alveolar bone grafting (ABG), age at speech assessment, sex, number of operations, pharyngoplasty, and ONF-related data.

Logistic regression models were used for the following dependent variables: speech intelligibility (rated on a 5-point scale converted to a 0/1 scale), hypernasality (rated on a 3-point scale converted to a 0/1 scale), hyponasality, various misarticulation patterns, and voice abnormalities. In each model, the independent variables included age at cleft repair, age at ABR measurement, age at speech assessment, pharyngoplasty, history of ONF closure, and presence of ONF.

## 3. Results

### 3.1. Sample

Ninety-three patients with UCLP were consecutively operated at IMC between 7 April 2005 and 14 March 2007. Fourteen of them were syndromic and four patients had incomplete UCLP—they were excluded from UCLP group. Additionally, 19 patients did not attend the speech evaluation at the age of 10. Consequently, the UCLP group consisted of 56 patients, including 7 patients with Simonart’s band. Of the UCLP group, 37 patients were boys (66.1%) and 19 were girls (33.9%). They were born between 27 September 2004 and 2 August 2006, and underwent one-stage primary cleft repair using the same surgical technique between the ages of 5 and 16 months (mean age at surgery: 8.1 months, SD 2.4). The surgeries were performed by three experienced surgeons with an annual workload of over 100 cleft operations. In 49 patients, an alveolar bone graft (ABG) procedure was carried out (7 patients had not yet undergone ABG at the time of the speech evaluation). The ABG technique described in [16] was utilized, involving the use of autogenous bone blocks harvested from the anterior part of the iliac crest. According to the IMC’s treatment protocol for UCLP, ABG was typically performed before the age of 6, preferably between 2 and 4 years of age. However, only 34 patients underwent ABG before the age of 6 (of which 29 had ABG before the age of 4). In our sample, the mean age at ABG was 5.5 years (SD = 3.4, range 1.9 to 11.5)—see Table 1.

The mean age at speech evaluation in the UCLP group was 10.5 years. Sixteen patients (28.6%) had ear ventilation tube (VT) insertion at least once, and some of them underwent the procedure multiple times (mean number: 2.4, range: 1–4). None of the patients had significant hearing loss. Two patients (3.6%) underwent upper pedicled flap pharyngoplasty due to inadequate speech improvement despite intensive speech therapy focused on velopharyngeal sphincter function. No patients were referred for other surgical interventions to improve velopharyngeal competence, such as sphincteroplasty or secondary palatoplasty. Six patients (10.7%) underwent ONF repair—three of them during ABG surgery, while three patients had independent ONF repair procedures. Additional information regarding the clinical characteristics of the groups is presented in Table 1.

### 3.2. Method Reliability

The lowest intra- and inter-rater reliability was observed for hypernasality (κ = 0.471) and voice abnormalities (κ = 0.412), respectively, indicating moderate agreement. The remaining intra- and inter-rater reliability determinants for the assessed speech parameters demonstrated very good and good agreement.

### 3.3. Speech Outcome

Table 2 and Table 3 present the speech outcomes in the UCLP and control groups. Speech intelligibility was poorer in the UCLP group compared to the control group (*p* = 0.001). Hypernasality, hyponasality, facial grimacing, and retracted articulation were exclusively observed in patients with UCLP. Other speech problems, such as voice abnormalities, misarticulated interdental sound, misarticulated addental sound, and misarticulated “r” sound, were present in both the UCLP and control groups. However, the only statistically significant difference between the two groups was observed in relation to voice abnormalities (*p* = 0.013).

Pairwise correlations, with a Bonferroni-adjusted significance level, revealed statistically significant associations among three parameters: speech intelligibility, hypernasality, and facial grimacing. The correlation coefficients (*p*-values) for intelligibility–hypernasality, intelligibility–grimacing, and hypernasality–grimacing were 0.84 (<0.001), 0.5 (0.012), and 0.49 (0.02), respectively. When the significance level was not adjusted, several other associations between the analyzed parameters were found (see Supplementary Table S1).

Regression analyses indicated that only speech intelligibility and the presence of hypernasality were predicted by independent variables (see Table 4 and Table S2). Age at UCLP repair and a positive history of ONF closure predicted speech intelligibility (*p* = 0.014 for the whole model), while age at UCLP repair, age at speech assessment, positive history of ONF closure, and the presence of ONF predicted hypernasality (*p* = 0.024 for the whole model). The timing of ABG had no effect on the dependent variables.

## 4. Discussion

The main objective of this study was to evaluate the speech outcomes and the need for secondary surgical interventions related to speech in patients with UCLP who underwent one-stage closure of the entire cleft using double-layer vomeroplasty to minimize palatal scarring. This technique allows for the significant reduction in or complete elimination of palatal scarring by utilizing palatal flap distraction in the hard palate area. Temporary postoperative palatal vault shallowing followed by recovery within a few days was observed as a result. The secondary aim of the study was to investigate the impact of different timings of alveolar bone grafting (ABG) between 2 and 11 years on speech development. To achieve these research goals, we utilized the perceptual speech assessment (PSA) recognized as the gold standard for appraising speech outcomes in individuals with cleft palate and velopharyngeal dysfunction [17].

Our findings suggest that the current protocol for treating 10-year-olds with UCLP leads to favorable speech outcomes. Approximately 78.5% of the subjects in our study demonstrated good or very good speech intelligibility, with 71.4% showing no signs of hypernasality and 87.5% exhibiting no hyponasality. In comparison, the results of the Scandcleft project [18,19], which encompassed three parallel randomized clinical controlled trials initiated in 1997 to investigate the relationship between different surgical protocols and treatment outcomes in cleft lip and palate, showed that 45–50% of 5-year-olds had no hypernasality, and approximately 50–60% demonstrated satisfactory velopharyngeal competency, with approximately 13% requiring pharyngoplasty, with significant differences between the trials. At 10 years, between 47% and 68% participants of the Scandcleft study demonstrated favorable velopharyngeal competence (hypernasality and hyponasality scores were not reported at 10 years). While our findings suggest that patients treated in Warsaw exhibited more favorable speech development compared to their counterparts in the Scandcleft project, it is crucial to consider the methodological differences between these two studies. The Scandcleft project has a longitudinal and prospective design, marked by comprehensive adherence and a minimal drop-out rate. In contrast, our investigation takes a retrospective approach and has encountered a notable rate of patient drop-out. It is worth noting that children with less favorable speech outcomes might not have been assessed due to their absence from routine checkups and periodic evaluations at IMC. These methodological variations are essential to bear in mind when interpreting the contrasts between our findings and those of the Scandcleft trial.

In our previous studies, we evaluated speech outcomes in patients with UCLP who underwent palatoplasty using previously employed techniques, such as von Langenback and single-layer vomerplasty [12,13]. Older reports showed that a small percentage of patients had mild to severe hypernasality (mean of three groups: 12.4%). However, in the current sample, 28.6% of 10-year-olds with UCLP exhibited some degree of hypernasality. The difference in hypernasality rates can be attributed to the challenges in assessing hypernasality, as the low reliability of assessments has been frequently reported in the literature [19,20,21]. Rating hypernasality also presented challenges in our study, as evidenced by low values of kappa for intra- and inter-rater agreement. On the other hand, the significantly lower rate of oronasal fistulas (ONFs) in the current group compared to our previous reports (7% vs. approximately 50%) suggests that the modification of the surgical technique by introducing double-layer closure for the hard palate resulted in a significant improvement in treatment outcomes.

We found that the timing of ABG had no discernible influence on speech development, as none of the evaluated speech parameters exhibited any correlation with the age at which the bone grafting procedure was executed. However, our clinical observations indicate that initiating alveolar surgical reconstruction at an earlier stage has demonstrated advantages for subsequent speech therapy. This advantage becomes particularly evident in the amelioration of tongue muscle activity, frequently leading to an enhanced correction of ‘s’ sound errors. It remains plausible that future investigations utilizing more refined methodologies could offer deeper insights into this facet of speech therapy. It is satisfying to observe that our surgical protocol, including early ABG, resulted in overall good speech outcomes, especially considering that early ABG is rarely practiced in cleft centers worldwide.

The secondary ABG, usually performed between the ages of 9 and 11 years has been recommended since the 1970s. This approach proved more successful in terms of subsequent maxillary growth compared to the previously used primary bone grafting, which was associated with detrimental effects on craniofacial growth. The optimal timing of secondary ABG gained prominence due to the negative reputation of primary ABG, typically performed before or at the time of primary cleft repairs. Consequently, the late timing of bone grafting became the preferred practice for most cleft teams worldwide [22]. However, recent long-term observations of maxillary growth [23] and alveolar bone volume [24] following bone grafting at different timings have challenged this conventional understanding. These studies have indicated that the inhibitory influence of the surgical procedure is not permanent; rather, its inhibitory effect remains active for a limited period, after which bone tissue seems to return to its normal growth trajectory. Consequently, the secondary alveolar bone grafting procedure can exert a similar influence on maxillary growth, regardless of its timing, as long as it avoids periods of intense growth, such as the prepubertal growth spurt or the first three years of life. Notably, growth intensity remains relatively consistent from the age of 3 until the prepubertal growth spurt. However, it is challenging to determine the extent to which early ABG contributed to favorable speech development because two factors were modified simultaneously in our sample—the surgical management of the cleft palate and the timing of ABG. Regardless, a comparison of the incidence of ONFs between previous reports and the current study shows significant improvement. Moreover, the ONFs in our sample were Veau III type and small in size, eliminating the need for extensive dissection during repair.

This study highlights that despite advanced surgical treatment and intensive speech therapy, there is still a noticeable difference in speech between children with and without UCLP. Velopharyngeal dysfunction, which is absent in children without UCLP and present in some UCLP patients, can result from various causes such as velopharyngeal mislearning, velopharyngeal incompetence, and velopharyngeal insufficiency [25]. The hypernasality commonly observed in UCLP patients is likely due to both anatomical and functional deficiencies. Therefore, reducing the occurrence of hypernasality in UCLP patients would require a close collaboration between cleft surgeons focusing on restoring pharyngeal structures and speech therapists aiming to improve abnormal function.

Middle ear diseases like otitis media with effusion (OME) are common in childhood [26]. However, children with CP ± L experience prolonged recovery and a significant incidence of late sequelae compared to children without cleft palate [27]. Ear infections and hearing impairment are particularly prevalent in 4–6-year olds with CP ± L and can persist at a substantial level for many years. These problems tend to settle only after the age of 12 years [27]. In our study, 28.6% of UCLP patients received ventilation tube (VT) insertion to alleviate the effects of OME, which is a high proportion compared to age-matched controls but consistent with the typical rates in patients with orofacial clefts. A recent systematic review demonstrated VT insertion in 38% to 53% of cleft patients with OME [28]. Thus, our results appear more favorable than those summarized in the mentioned systematic review. The early timing of primary UCLP repair in our sample (mean 8.1 months) may have contributed to the relatively low incidence of VT insertion, as early palatal surgery is presumed to reduce the tendency to develop OME [6,29].

## 5. Limitations

In the field of cleft research, retrospective studies predominate due to the considerable time lapse between an event, such as a surgical operation, and the subsequent effects on factors like speech or growth. However, the retrospective design introduces the potential for bias, particularly in the form of selection bias. This occurs when only specific patients are included, potentially leading to skewed outcomes. To address this concern, researchers aim to include consecutively treated patients in their studies. Nonetheless, assessing all patients who were consecutively operated on a decade earlier presents challenges, as well as, in the case of this investigation, due to factors like relocations, distances to cleft centers, and individual patient preferences, all of which can influence participation.

It is noteworthy that the missing data likely arose randomly, which could have minimized any potential bias in patient selection and its impact on the obtained results. For instance, it is equally plausible that a patient who did not attend periodic evaluations did so out of dissatisfaction with the treatment or because they were content with the results, seeing no need for the proposed treatment and subsequent evaluation.

## 6. Conclusions

In general, 10-year-old individuals with unilateral cleft lip and palate who followed the treatment protocol involving one-stage closure of the entire cleft and early secondary alveolar bone grafting demonstrated speech irregularities that are frequently observed within this patient population. However, the prevalence of significant speech abnormalities, such as moderate or severe hypernasality, was relatively low and in line with other successful treatment approaches. Furthermore, there appeared to be a relatively low requirement for surgical interventions aimed at addressing speech-related concerns.

## Figures and Tables

**Table 1:** Group characteristics.

	UCLP					Control					*p* Value
	Mean	SD	Median	25%	75%	Mean	SD	Median	25%	75%	
Age at 1-stage repair of cleft (months)	8.1	2.4	7.3	6.6	8.8	n/a					
Age at ABG (years)	5.5	3.4	3.9	2.7	9.8	n/a					
Age at speech assessment (years)	10.5	0.6	10.6	10.1	11.0	9.9	0.6	10	9.3	10	<0.001
Fistula present/absent	0.1	0.3	0	0	0	n/a					
Fistula repair performed	0.1	0.3	0	0	0	n/a					
Pharyngoplasty performed	0	0.2	0	0	0	n/a					
Number of operations (primary and secondary)	2.6	0.6	2	3	3	n/a					
% Cleft side: right/left	32.5%/67.5%					n/a					
Simonart’s band present	7 (12.5%)					n/a					
Ventilation tube insertion	16 (28.6%)					n/a					

SD—standard deviation; n/a—not applicable.

**Table 2:** Speech abnormalities in the study and control groups.

	UCLP					Control					*p* Value	Type of the Test
	Mean	SD	Median	25%	75%	Mean	SD	Median	25%	75%		
intelligibility	1.9	0.9	2	1	2	1.4	0.5	1	1	2	0.001	Wilcoxon test
hypernasality	0.5	1.0	0	0	1	0.0	0.0	0	0	0	<0.001	Wilcoxon test
hyponasality	0.1	0.3	0	0	0	0.0	0.0	0	0	0	0.001	test of proportions
grimacing	0.1	0.4	0	0	1	0.0	0.0	0	0	0	0.055	Wilcoxon test
voice abnormalities	0.2	0.4	0	0	0	0.0	0.1	0	0	0	0.013	test of proportions
mis_inter	0.3	0.4	0	0	0	0.4	0.5	0	0	1	0.149	test of proportions
mis_ad	0.1	0.2	0	0	0	0.0	0.2	0	0	0	0.742	test of proportions
retracted	0.1	0.3	0	0	0	0.0	0.0	0	0	0	0.017	test of proportions
mis_r	0.2	0.4	0	0	0	0.1	0.3	0	0	0	0.284	test of proportions

mis_inter—misarticulated interdental sound; mis_ad—misarticulated addental sound; mis_r—misarticulated “r” sound; SD—standard deviation.

**Table 3:** Detailed data regarding speech, surgical, and morphological parameters.

		UCLP		Control	
	Grade	Frequency	%	Frequency	%
Intelligibility	1	19	33.9	29	58
	2	25	44.6	21	42
	3	9	16.1	0	0
	4	2	3.6	0	0
	5	1	1.8	0	0
Hypernasality	0	40	71.4	50	100
	1	6	10.7	0	0
	2	7	12.5	0	0
	3	2	3.6	0	0
	4	1	1.8	0	0
Hyponasality	0	49	87.5	50	100
	1	7	12.5	0	0
Facial grimacing	0	52	92.9	50	100
	1	2	3.6	0	0
	2	2	3.6	0	0
Voice abnormalities	0	47	83.9	49	98
	1	9	16.1	1	2
Misarticulated interdental sound	0	41	73.2	30	60
	1	15	26.8	20	40
Misarticulated addental sound	0	53	94.6	48	96
	1	3	5.4	2	4
Retracted sounds	0	50	89.3	50	100
	1	6	10.7	0	0
Misarticulated “r” sound	0	45	80.4	44	88
	1	11	19.6	6	12
Fistula presence: yes/no	0	52	92.9	n/a	
	1	4	7.1		
Fistula closure	0	51	91.1	n/a	
	1	5	8.9		
Pharyngoplasty performed	0	54	96.4	n/a	
	1	2	3.6		
Number of operations	2	25	44.6	n/a	
	3	26	46.4		
	4	5	8.9		

**Table 4:** Regression models.

Independent Variables	Odds Ratio	SE	*p* Value	95% CI Lower Limit	95% CI Upper Limit	Summary of the Model
Dependent variable: **intelligibility**						
Age at primary repair (months)	0.52	0.16	**0.038**	0.29	0.96	N = 54 *p* = 0.014 Pseudo-R^2^ = 0.276
Age at alveolar bone grafting	1.15	0.17	0.356	0.86	1.54	
Age at assessment	6.75	7.08	0.069	0.86	52.82	
Pharyngoplasty (yes/no)	*1.00*	*(omitted)*				
Fistula closure (yes/no)	23	31.08	**0.020**	1.63	325.19	
Fistula presence (yes/no)	7.17	11.34	0.213	0.32	159.03	
Dependent variable: **hypernasality**						
Age at primary repair (months)	0.63	0.14	**0.037**	0.41	0.97	N = 54 *p* = 0.024 Pseudo-R^2^ = 0.209
Age at alveolar bone grafting	1.21	0.15	0.141	0.94	1.55	
Age at assessment	6.35	5.73	**0.041**	1.08	37.27	
Pharyngoplasty (yes/no)	*1.00*	*(omitted)*				
Fistula closure (yes/no)	13.00	15.78	**0.035**	1.20	140.40	
Fistula presence (yes/no)	13.43	17.68	**0.048**	1.02	177.22	

## Data Availability

Not applicable.

## Supplementary Materials

The following supporting information can be downloaded at: https://www.mdpi.com/article/10.3390/jcm12175545/s1, Table S1. Pairwise correlations; Table S2. Regression models.
