# Supplementary material for: Speech and Burden of Secondary Surgical Interventions Following One-Stage Repair of Unilateral Cleft Lip and Palate and Alveolar Bone Grafting Performed at Different Timings
Source: J Clin Med. 2023 Aug 25;12(17):5545. doi: 10.3390/jcm12175545 (PMC10489147; doi:10.3390/jcm12175545)
Supplement: Supplementary file 1 [file jcm-12-05545-s001.zip › jcm-2559450-supplementary.pdf]

Supplementary Table S1. Pairwise correlations

	intel	hyper	hypo	grimacing	v_abnor	mis_inter	mis_ad	retr	mis_r	fist_y_n	fist_clos	phar	No_oper	sex	age_rep	age_abg	age_spe
**intel**	x	0.84 (<0.001)		0.5 (<0.001)				0.28 (0.037)	0.38 (0.004)			0.33 (0.012)					
**hyper**	0.84 (<0.001)	x		0.49 (<0.001)				0.35 (0.009)				0.39 (0.003)					
**hypo**			x														
**grimacing**	0.5 (<0.001)	0.49 (<0.001)		x			0.33 (0.012)										
**v_abnor**					x												
**mis_inter**						x	0.39 (0.003)										
**mis_ad**				0.33 (0.012)		0.39 (0.003)	x										
**retr**	0.28 (0.037)	0.35 (0.009)						x			0.30 (0.027)						
**mis_r**	0.38 (0.004)								x								
**fist_y_n**										x							
**fist_clos**								0.30 (0.027)			x		0.37 (0.005)				
**phar**	0.33 (0.012)	0.39 (0.003)										x					
**No_oper**											0.37 (0.005)		x				
**sex**														x			
**age_rep**															x	0.40 (0.002)	
**age_abg**															0.40 (0.002)	x	
**age_spe**																	x

Intel—intelligibility; hyper—hypernasality; hypo—hyponasality; grimacing—facial grimacing; v_abnor—voice abnormalities; mis_inter—misarticulated interdental sounds; mis_ad—misarticulated addental sounds; retr—retracted sounds; mis_r—misarticulated “r” sounds; fist_y_n— fistula presence, yes/no; fist_clos—fistula closure, yes/no; phar—pharyngoplasty performed, yes/no; No_oper—number of primary and secondary operations; age_rep—age at cleft repair; age_abg—age at alveolar bone grafting; age_spe—age at speech assessment.

Supplementary Table S2. Regression models

Independent variables	Odds ratio	SE	P value	95% CI lower limit	95% CI upper limit	Summary of the model
Dependent variable: **hyponasality**						
Age at primary repair (months)	0.89	0.19	0.59	0.58	1.37	N = 54<br>P = 0.162<br>Pseudo-R^2 = 0.19
Age at alveolar bone grafting	1.04	0.17	0.82	0.75	1.43	
Age at assessment	8.78	10.81	0.08	0.78	98.17	
Pharyngoplasty (yes/no)	1.00	(omitted)				
Fistula closure (yes/no)	15.05	20.30	0.04	1.07	211.46	
Fistula presence (yes/no)	14.93	24.85	0.10	0.57	389.56	
Dependent variable: **voice abnormalities**						
Age at primary repair (months)	1.45	0.28	0.06	0.99	2.11	N = 56<br>P = 0.345<br>Pseudo-R^2 = 0.137
Age at alveolar bone grafting	0.77	0.13	0.11	0.56	1.06	
Age at assessment	0.94	0.76	0.94	0.19	4.57	
Pharyngoplasty (yes/no)	3.64	5.46	0.39	0.19	68.66	
Fistula closure (yes/no)	1.17	1.66	0.91	0.07	18.87	
Fistula presence (yes/no)	2.72	3.65	0.46	0.20	37.84	
Dependent variable: **misarticulated interdental sounds**						
Age at primary repair (months)	0.97	0.15	0.83	0.71	1.31	N = 49<br>P = 0.985<br>Pseudo-R^2 = 0.006
Age at alveolar bone grafting	1.06	0.11	0.60	0.86	1.29	
Age at assessment	0.98	0.59	0.97	0.30	3.16	
Pharyngoplasty (yes/no)	1.00	(omitted)				
Fistula closure (yes/no)	1.00	(omitted)				
Fistula presence (yes/no)	0.74	0.92	0.81	0.07	8.35	
Dependent variable: **misarticulated addental sounds**						
Age at primary repair (months)	0.65	0.28	0.32	0.27	1.53	N = 45<br>P = 0.559<br>Pseudo-R^2 = 0.093
Age at alveolar bone grafting	1.25	0.24	0.25	0.86	1.83	
Age at assessment	1.42	1.71	0.77	0.13	15.10	
Pharyngoplasty (yes/no)	1.00	(omitted)				
Fistula closure (yes/no)	1.00	(omitted)				
Fistula presence (yes/no)	1.00	(omitted)				
Dependent variable: **retracted sounds**						
Age at primary repair (months)	0.99	0.21	0.98	0.65	1.52	N = 54<br>P = 0.386
Age at alveolar bone grafting	1.06	0.17	0.72	0.77	1.46	

Age at assessment	2.55	2.93	0.41	0.27	24.14	Pseudo-R^2 = 0.14
Pharyngoplasty (yes/no)	1.00	(omitted)				
Fistula closure (yes/no)	13.43	16.69	0.04	1.17	153.52	
Fistula presence (yes/no)	8.51	13.10	0.16	0.42	174.10	
Dependent variable: **misarticulated "r" sounds**						
Age at primary repair (months)	1.11	0.21	0.59	0.76	1.61	N = 47<br>P = 0.529<br>Pseudo-R^2 = 0.062
Age at alveolar bone grafting	0.82	0.11	0.15	0.63	1.07	
Age at assessment	0.87	0.56	0.83	0.24	3.10	
Pharyngoplasty (yes/no)	2.59	3.89	0.53	0.14	49.02	
Fistula closure (yes/no)	1.00	(omitted)				
Fistula presence (yes/no)	1.00	(omitted)				
